# ﻿*Thismiakenyirensis* (Thismiaceae), a new species from Taman Negeri Kenyir, Terengganu, Peninsular Malaysia

**DOI:** 10.3897/phytokeys.221.98571

**Published:** 2023-03-07

**Authors:** Mat Yunoh Siti-Munirah, Nikong Dome

**Affiliations:** 1 Forest Research Institute Malaysia, 52109 Kepong, Selangor, Malaysia Forest Research Institute Malaysia Kepong Malaysia; 2 DigitalDome Photography, 21500 Permaisuri, Terengganu, Malaysia DigitalDome Photography Terengganu Malaysia

**Keywords:** *Brunonithismia*, lowland dipterocarp forest, rare species

## Abstract

A new mycoheterotrophic species, *Thismiakenyirensis* Siti-Munirah & Dome from Peninsular Malaysia, is described and illustrated. *Thismiakenyirensis* differs from other previously described species in the following characteristics: the flower tube is completely orange, with alternating darker and paler-coloured longitudinal lines on the outer and inner surfaces, the outer tepals are ovate (petaloid), the inner tepals are narrowly lanceolate, each ending with a long appendage. According to the IUCN Red List categories and criteria, *T.kenyirensis* is provisionally classified as Least Concern.

## ﻿Introduction

*Thismia* Griff. is a genus of non-photosynthetic flowering plants belonging to the family Thismiaceae. *Thismia* species are small herbs, with scale-like leaves and actinomorphic or zygomorphic, urceolate to campanulate flowers. *Thismia* includes about 100 species worldwide (from Imhof 2010 onwards, Siti-Munirah et al. 2022) and it is the most widespread and species-rich genus in the family Thismiaceae with high endemism. Their range extends from tropical and subtropical Asia to northern and eastern Australia and New Zealand and from tropical South America to Costa Rica, with isolated occurrence in North America (POWO 2022). Many species are believed to be extremely rare and have a scattered range. They are easily overlooked in the wild due to their small size and the short-lived nature of their aboveground parts (Larsen and Averyanov 2007). In Malaysia, Terengganu is currently the state with the most *Thismia* records, with 11 reported species: *T.alba* Holttum ex Jonker, *T.arachnites* Ridl, *T.aseroe* Becc, *T.brunneomitroides* Suetsugu & Tsukaya, *T.domei* Siti-Munirah, *T.clavigeroides* Chantanaorr. & Seelanan, *T.javanica* J.J.Sm., *T.latiffiana* Siti-Munirah & Dome, *T.ornata* Dančák, Hroneš & Sochor, *T.sitimeriamiae* Siti-Munirah, Dome & Thorogood, *T.terengganuensis* Siti-Munirah (Siti-Munirah and Dome 2019, 2022a, b; Siti-Munirah et al. 2021).

**Map 1.**
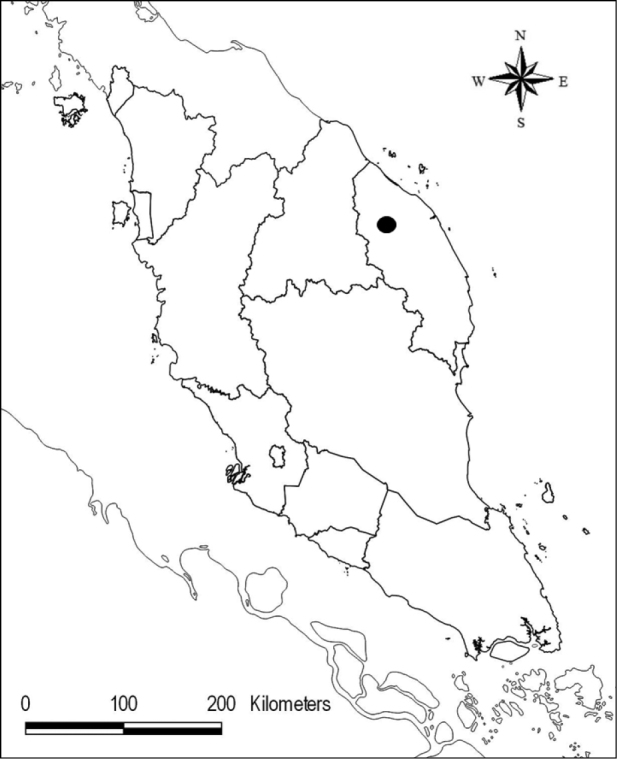
Taman Negeri Kenyir, Terengganu (black circle), the type locality of *Thismiakenyirensis*.

**Map 2.**
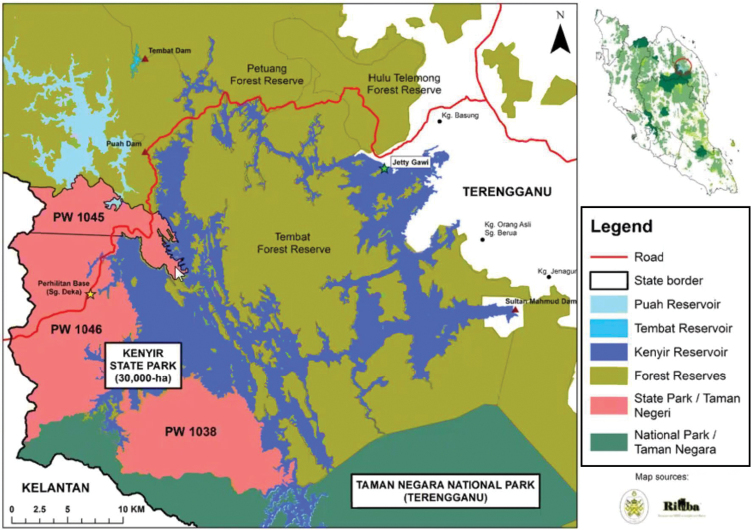
Map of Tasik Kenyir and the forest boundaries. (Map sourced from NBS).

Currently, five of the species reported in Terengganu are known from the Kenyir State Park (Taman Negeri Kenyir, TNK) (Siti-Munirah and Dome 2022b). The TNK is located in the eastern part of Peninsular Malaysia in the state of Terengganu (Map 1). It used to be part of the Tembat Forest Reserve (FR), but in 2018 and 2019, parts of Tembat FR were designated as TNK. The forest area includes part of the forest around Tasik Kenyir (Kenyir Lake), which is adjacent to Terengganu National Park (Taman Negara Terengganu) and also connected to Tembat FR forest and Kelantan State (Map 2). During the scientific expedition to the TNK on 8 September 2020, the second author and his team visited the Sungai Cendana area in the southern part of the TNK and discovered a population of an unknown *Thismia* plant, which he forwarded to the first author. Together, we revisited the populations in June 2022. After further investigation and comparison with known species of *Thismia*, we concluded that this plant is distinct from previously described species of the genus. We therefore report and describe it here as a new species for science.

## ﻿Materials and methods

Our assessment is based on material collected in Taman Negeri Kenyir, Hulu Terengganu, Terengganu (Map 1). The specimens were preserved in 70% ethanol. All the specimens have been deposited in Kepong Herbarium (**KEP**) and have been examined for taxonomic treatment. Morphological characters and measurements were examined using an Olympus SZ61 stereo microscope and high-resolution macrophotography. Additionally, measurements were taken from spirit material. The specimen features were compared in detail with the original drawings and descriptions of morphologically similar species of *Thismia* provided by Jonker (1938), Larsen and Averyanov (2007), Mar and Saunders (2015), Chantanaorrapint (2018), Tanaka et al. (2018), Suetsugu et al. (2018), Siti-Munirah and Dome (2019) and Nuraliev et al. (2020).

## ﻿Taxonomic account

### ﻿ Thismia kenyirensis

Taxon classificationPlantaeDioscorealesThismiaceae

Siti-Munirah & Dome sp. nov.

A9675D79-5A92-5D66-B145-4F4E197D535E

urn:lsid:ipni.org:names:77315245-1

Figs 1 , 2 , 3

#### Diagnosis.

Distantly similar to *Thismiahongkongensis* Mar & Saunders but differs by petaloid, ovate outer tepals which are ca. 8 mm long, narrowly lanceolate-linear, ca. 8 mm long inner tepals which are not forming a lose-dome, up to 28 mm long appendages of inner tepals, orange floral tube, which lacks reticulate pattern on its inner surface and connective apex with 3 long appendages.

**Figure 1.**
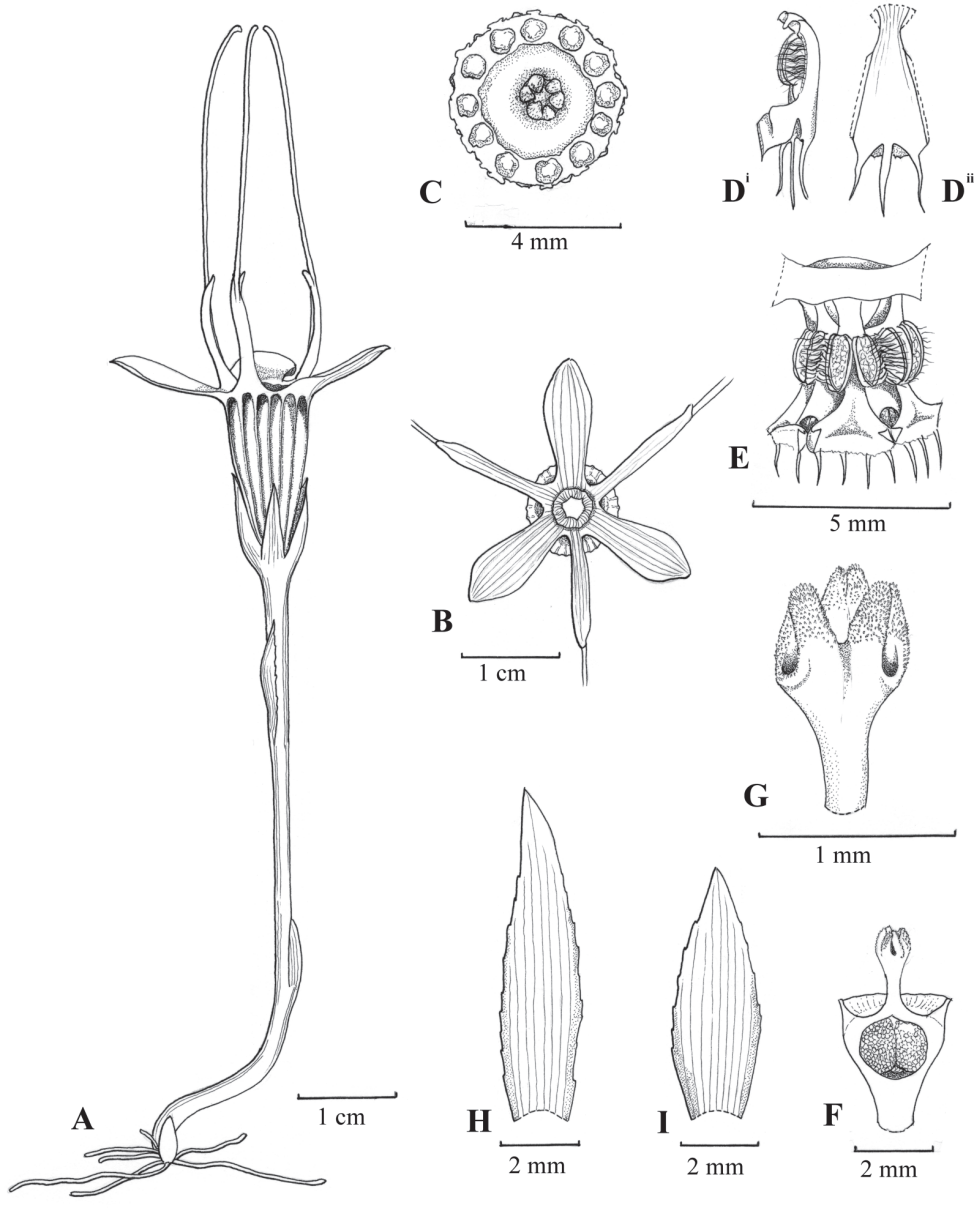
*Thismiakenyirensis***A** plant with flower and roots **B** top view of flower showing tepals **C** top view of ovary and stigma **D** stamen (i, side view; ii, inner view) **E** outer view of three pendulous stamens **F** longitudinal section of ovary **G** style and stigma **H** bract **I** leaf. All from FRI91122a (spirit material). Drawings by Mohamad Aidil Noordin.

#### Type.

Malaysia. Peninsular Malaysia: Terengganu, Hulu Terengganu District, Taman Negeri Kenyir, Sungai Cendana, elev. ca 204 m, 8 Sept 2020, Dome Nikong, FRI 91122a (holotype KEP!, spirit collection, barcode no. SC12015).

**Figure 2.**
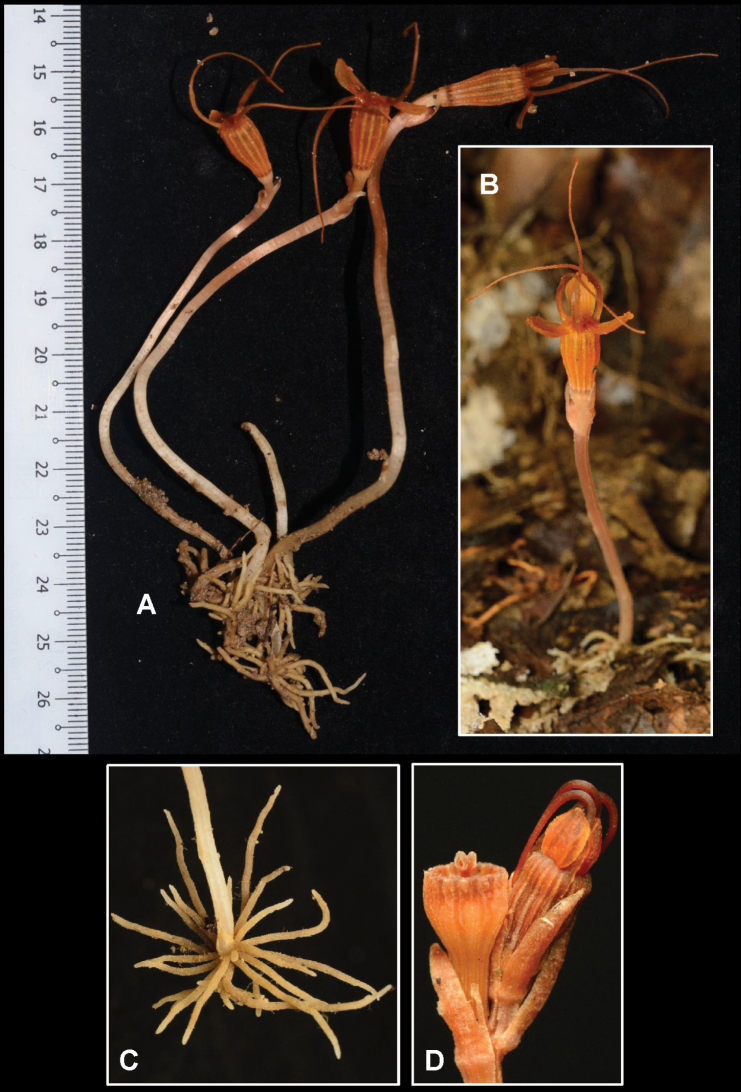
*Thismiakenyirensis***A** mature plant with flowers and roots, scale in centimetres (cm) (FRI91122b) **B** mature plant in natural habitat (FRI91122a) **C** vermiform roots (FRI91122c) **D** young flower with mature ovary and stigma (FRI91122c). Photos by Siti-Munirah MY (**A**); Dome Nikong (**B–D**).

#### Description.

Achlorophyllous herb, up to 130 mm tall. ***Roots*** vermiform, unbranched, ca. 1 mm in diameter, light brown. ***Stem*** erect, up to 90 mm long, 1.5–2 mm in diameter, pale brownish-orange, bearing 1–2 flowers. ***Leaves*** 2–4, spirally arranged, triangular to narrowly triangular, scale-like, acute, margin almost entire but slightly irregularly serrate, 2–6 mm long, ca. 1 mm wide at base, pale brown. ***Involucral bracts*** 3, similar to leaves but slightly larger, spirally arranged, triangular to narrowly triangular, scale-like, acute, entire, 8 mm long, ca. 1.5–2.5 mm wide at base, pale brown or pale orange. ***Pedicel*** up to 6 mm long at anthesis, elongating to ca. 10 mm long after anthesis, pale brown. ***Flowers*** terminal, actinomorphic, ca. 52 mm long (including ovary, floral tube and inner tepal with appendage); ***floral tube*** urceolate, 12–15 mm long, ca. 5 mm wide at base, ca. 6 mm wide at middle, ca. 7 mm wide distally; ***outer surface*** glabrous, orange to brownish-orange, with 12 darker orange longitudinal ribs alternating with 12 paler longitudinal lines, ***inner surface*** smooth or rough, almost similar to outer surface, without transverse bars or reticulate ornamentation; ***outer tepals*** 3, free, petaloid, ovate, apex acute, 8 mm long, ca. 4–5.5 mm wide (ca. 4 mm at base, ca. 5.5 mm above middle), glabrous, bright orange; ***inner tepals*** 3, free, narrowly lanceolate-linear, 8 mm long, ca. 1 mm wide, glabrous, dark orange, apically bearing a tentacle-like appendage; appendage narrowing towards apex, ca. 27–28 mm long, 0.5 mm wide, dark orange at base and bright orange at apex; ***annulus*** dark orange, glabrous. ***Stamens*** 6, pendent from the annulus, outer side greenish-orange or greenish-brown, inner side dark-brown to blackish and paler, ***filaments*** free, ca. 1 mm long, curved downwards; ***connectives*** flattened at inner surface, laterally connate to form a tube, ca. 4–5 mm long, narrow at base (ca. 0.8 mm wide) and broad at apex (ca. 1.7 mm wide), connective apex with 3 appendages, slightly curved inside, each appendage ca. 1.5 mm long; outer side of connective bearing a skirt-like lateral appendage protruding towards floral tube; lateral appendage not exceeding the tip of the connective appendages, with short translucent trichomes on margin, ***interstaminal glands*** greenish translucent, inserted on the line of fusion between connectives at the level of attachment of lateral appendages. ***Ovary*** inferior, unilocular; ***placentas*** 3, free, forming columns and arising from the bottom of the ovary; ovules numerous. ***Style*** dark brown-orangish, ca. 1 mm long; ***stigma*** ca. 1 mm long, papillose, 3-lobed, with lobes ± rectangular, bifurcate at apex, pale brown or orangish to whitish. ***Fruit*** cup-shaped, 3–5 mm in height, 4–6 in diameter, pale orange (or white to creamy orange), darker at upper part. ***Seeds*** unknown.

**Figure 3.**
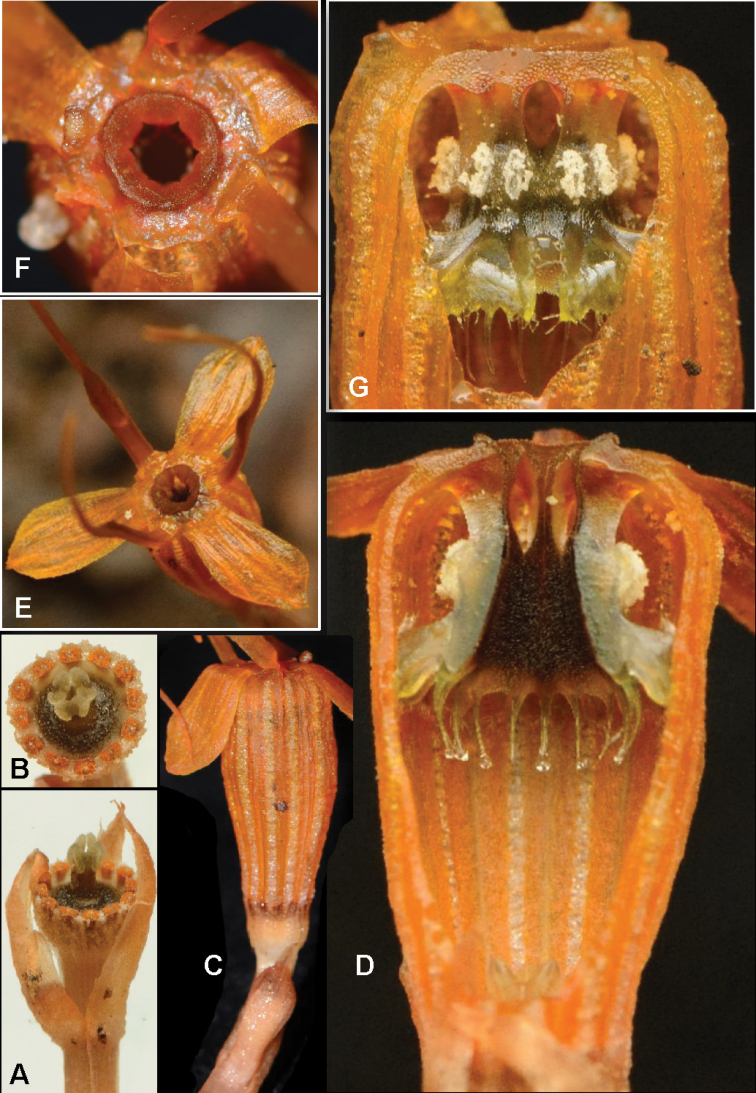
*Thismiakenyirensis***A** bracts with ovary and pistil, lateral view **B** top view of stigma **C** flower, side view **D** longitudinal section of floral tube and stamens **E** flower, top view **F** annulus **G** stamens, outer view. Photos by Siti-Munirah MY (**A–C, E, F**; All from FRI 91122b); Dome Nikong (**D, G**; No specimens).

#### Additional specimen examined (paratype).

Malaysia. Peninsular Malaysia: Terengganu, Hulu Terengganu District, Taman Negeri Kenyir, Sungai Cendana, elev. ca 204 m, 8 Sept 2020, Dome Nikong, FRI 91122b (KEP, spirit collection, barcode no. SC12016), FRI 91122c (KEP, spirit collection, barcode no. SC12017); Taman Negeri Kenyir, Sungai Cendana, elev. ca. 156 m, 16 June 2022, Siti-Munirah MY, FRI98678 (KEP, spirit collection, barcode no. SC12018).

#### Distribution.

Endemic to Terengganu, Peninsular Malaysia. Currently, the occurrence is only from the type locality, Sungai Cendana area (Map 1). In addition, *T.kenyirensis* was observed in two other populations in the vicinity of Tasik Kenyir. It was sighted near Sungai Saok and on the way to Gunung Gagau (Wong Pui May personal communication), both in the Tasik Kenyir region. However, there are still no specimens and GPS information to verify these findings. This means that only the occurrence of the population in Sungai Cendana has been confirmed.

#### Ecology.

In moist shady areas of lowland dipterocarp forest on moist soil at elevations of 150–220 m a.s.l. Flowering and fruiting mostly from September to April, but also recorded to flower in June. Historically, the type locality was botanized as early as 2007. Based on the results of Lim et al. (2008), *T.alba* was also recorded during the survey in the Sungai Cendana area. This is in contrast to *T.kenyirensis*, which was not mentioned, implying that it was not seen or collected at that time. During our recent visit, we were also able to find *T.alba* not far from the original population of *T.kenyirensis*.

#### Etymology.

The epithet refers to Lake Kenyir, Kenyir State Park (Taman Negeri Kenyir), where the species was found.

#### Conservation status.

According to the IUCN standards (IUCN 2022), we propose to classify the preliminary conservation status of the species as Least Concern (LC). This is because the species is likely to be widespread in TNK, where it has been sighted a few times at the type locality (Sungai Cendana) and also by other observers at two other sites, and its entire habitat is in a fully protected forest area. In addition, nearby forests in Terengganu National Park (Taman Negara Terengganu) are also fully protected. As mentioned earlier, it is very likely that there are other populations in the vicinity of Tasik Kenyir. According to the second author’s observations, four individuals were sighted near Sungai Saok and one on the way to Gunung Gagau on 22 November 2022. However, no specimens are available for confirmation. *Thismiakenyirensis* occurs in intact habitats in forested areas in TNK. However, the species is very common only during the flowering season and its populations are mostly scattered, as few individuals or solitary individuals were observed during the flowering season. During the first check of the type locality, the population in Sungai Cendana in 2020, about 20 adult individuals were found. However, during a revisit in 2022, only a single individual was observed.

#### Notes.

**Figure 4.**
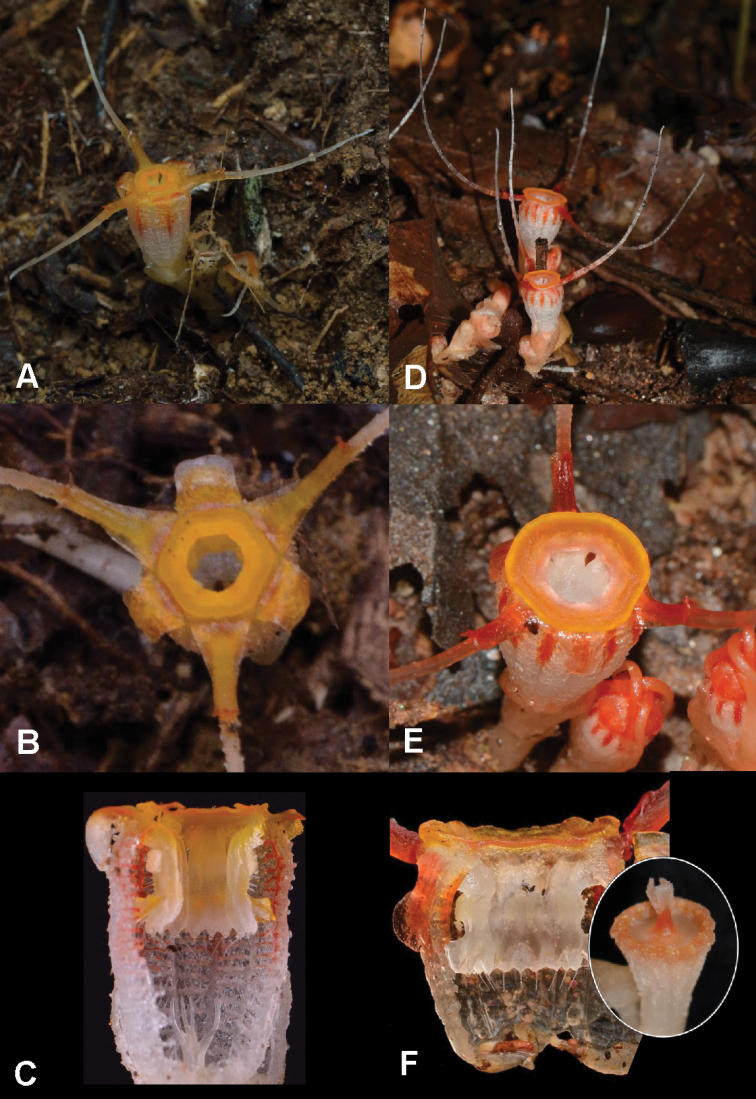
Morphologically close species to *T.kenyirensis* in Peninsular Malaysia **A–C***T.arachnites* (Taman Negeri Kenyir) **D–F***T.javanica* (Telemong Forest Reserve). Photos by Dome Nikong (**A–C, F**) and Siti-Munirah MY (**D, E, G**).

*Thismiakenyirensis* is easily recognised by the following combination of characteristics: vermiform roots, almost uniform light-to-dark orange flower coloration, petaloid ovate outer tepals, narrowly lanceolate-linear inner tepals with long appendages and each stamen with 3 appendages at its apex. Within the infrageneric classification by Kumar et al. (2017), *T.kenyirensis* resembles species of ThismiasubgenusThismia section ThismiasubsectionBrunonithismia Jonker, especially by its free and unequal tepals. Eight species are placed in subsectionBrunonithismia by Kumar et al. (2017): *T.arachnites*, *T.brunonis* Griff, *T.gardneriana* Hook.f. ex Thwaites, *T.hongkongensis*, *T.javanica*, *T.labiata* J.J.Sm., *T.neptunis* Becc. (proved to be only distantly related to the other species of the subsection by Shepeleva et al. 2020) and *T.tentaculata* K.Larsen & Aver. Of them, *T.arachnites* and *T.javanica* occur in Peninsular Malaysia, including the state of Terengganu. They both are readily distinguished from *T.kenyirensis* (Fig. 4, Table 1). In addition, *T.breviappendiculata* Nob. Tanaka (Tanaka et al. 2018) from Myanmar, *T.bokorensis* Suetsugu & Tsukaya (Suetsugu et al. 2018) from Cambodia, *T.gardneriana* from Sri Lanka and *T.tentaculata* from Hong Kong and Vietnam are similar to *T.kenyirensis*. In a molecular phylogenetic reconstruction provided by Shepeleva et al. (2020), *T.javanica*, *T.hongkongensis*, *T.gardneriana* and *T.tentaculata* occupied a relatively isolated and unstable position. All these species (and also *T.kenyirensis*) are characterised by free appendaged inner tepals and outer tepals without appendages. A more detailed phylogenetic analysis is required to test the monophyly of this morphological group, and the relationships of each of these species including *T.kenyirensis*.

**Table 1.** Morphological comparison of *T.kenyirensis* with similar species *T.arachnites* (Chantanaorrapint 2018), *T.bokorensis* (Suetsugu et al. 2018), *T.breviappendiculata* (Tanaka et al. 2018), *T.brunonis* (Jonker 1938), *T.gardneriana* (Jonker 1938; Bandara et al. 2020), *T.hongkongensis* (Mar et al. 2015), *T.javanica* (Siti-Munirah and Dome 2019; Nuraliev et al. 2020) and *T.tentaculata* (Larsen and Averyanov 2007; Nuraliev et al. 2020).

Characters	*T.kenyirensis*	*T.arachnites*	*T.bokorensis*	*T.breviappendiculata*	*T.brunonis*	*T.gardneriana*	*T.hongkongensis*	*T.javanica*	*T.tentaculata*
**Colour of floral tube**	Orange to brownish-orange with 12 darker orange longitudinal ribs	White to orange with 6 or 12 reddish vertical streaks in the upper part	Pure white	White, with 12 translucent longitudinal ridges	Yellowish	Yellowish-orange	Pinkish-white with 12 dark red vertical ribs	White to pale orange or light orangish-red	Pure white, sometimes with antetepalous veins indistinctly tinged with red
**Presence of transverse bars/ ornamentation on inner side of the floral tube**	Absent/absent	Present/absent	Absent/absent	Present/absent	Present/absent	Absent/ Not known	Absent/present	Present/absent	Absent/present
**Outer tepal Shape**	Ovate, petaloid	Broadly ovate	Broadly triangular, apex broadly obtuse or rounded	Ovate, obtuse	Broadly ovate, obtuse	Broad, rotundate	Triangular	Ovate-triangular	Isosceles triangular, broadly obtuse or rounded
**Length (mm)**	8	5–6.5	1.2	2	Not known	1.5	1.8	1–3.5	1.8–2.4
**Colour**	Bright orange	Yellow to orange.	Light yellow	Yellow	Not known	Yellow	Dark red	Orange to red or yellow, translucent	Light yellow or tinged with red, translucent
**Inner tepal (without appendage) Shape**	Narrowly lanceolate-linear	Triangular	Narrowly triangular	Isosceles triangular	Triangular at the base, caudate in long, thick, filiform tails	Subulate	Spatulate, adaxially concave	Narrowly triangular	Triangular with broad base
**Length (mm)**	8	6.5–9	2.6	2	Not known	Not known	3.3	1.5–3.3	
**Colour**	Bright orange	Orange to reddish-orange at the base	Light yellow	Yellow	Not known	Yellowish-orange	Dark red	Orange to red or yellow and usually dark red towards apex	Light yellow
**Stamen Apex**	3 long, slightly curved appendages	3-toothed, each tooth bearing a distinct stiff hair	3-toothed, each tooth narrowly triangular, with ca. 1 mm long	Lobed	With numerous teeth	2-toothed, each tapering into a stiff hair	2-toothed, adorned with trichomes	3-toothed, each tooth bearing hair	2-toothed, each tooth tapering into hair, sometimes with additional hairs between teeth

## Supplementary Material

XML Treatment for Thismia kenyirensis
